# Spermine Differentially Refines Plant Defense Responses Against Biotic and Abiotic Stresses

**DOI:** 10.3389/fpls.2019.00117

**Published:** 2019-02-08

**Authors:** Hamed Soren Seifi, Barry J. Shelp

**Affiliations:** Department of Plant Agriculture, University of Guelph, Guelph, ON, Canada

**Keywords:** abiotic stress, biotic stress, defense response, defense activator, signaling, spermine

## Abstract

Roles of the major polyamines (mPA), putrescine, spermidine, and spermine (Spm), in various developmental and physiological processes in plants have been well documented. Recently, there has been increasing focus on the link between mPA metabolism and defense response during plant-stress interactions. Empirical evidence is available for a unique role of Spm, distinct from the other mPA, in eliciting an effective defense response to (a)biotic stresses. Our understanding of the precise molecular mechanism(s) by which Spm modulates these defense mechanisms is limited. Further analysis of recent studies indicates that plant Spm functions differently during biotic and abiotic interactions in the regulation of oxidative homeostasis and phytohormone signaling. Here, we summarize and integrate current knowledge about Spm-mediated modulation of plant defense responses to (a)biotic stresses, highlighting the importance of Spm as a potent plant defense activator with broad-spectrum protective effects. A model is proposed to explain how Spm refines defense mechanisms to tailor an optimal resistance response.

## Introduction

Polyamines are ubiquitous, small aliphatic polycations found in eukaryotic organisms. The major polyamines (mPA) in plants are the diamine putrescine (Put), the triamine spermidine (Spd) and the tetraamine spermine (Spm). They function in key developmental and physiological events such as embryogenesis, cell division, floral initiation, senescence and responses to stress (Evans and Malmberg, 1989; Galston and Sawhney, 1990). The biosynthesis and degradation of mPA are highly responsive to environmental stimuli (Liu et al., 2007). Several studies have reported that the three mPA mold plant responses to (a)biotic stresses (Bouchereau et al., 1999; Walters, 2000, 2003a,b; Urano et al., 2003; Alcázar et al., 2010; Minocha et al., 2014; Romero et al., 2018). However, there is evidence for the differential regulation of Spm/Spd and Put by stresses (see Shelp et al., 2018), and for a unique role of Spm, distinct from the other mPA, in the induction and formation of resistance responses to various types of (a)biotic stresses. For instance, Mitsuya et al. (2009) reported that Spm is the only mPA that effectively suppresses the multiplication of cucumber mosaic virus in Arabidopsis. Other research indicates that Spm strongly induces different defense-related genes in Arabidopsis seedlings, whereas similar doses of Put and Spd do not, and elevated levels of endogenous Spm are causally linked to higher tolerance to the bacterial pathogen *Pseudomonas syringae* and the oomycete *Hyaloperonospora arabidopsidis* (Marco et al., 2014). Similarly, among the mPA, only Spm strongly induces the two key defense-associated signaling molecules, nitric oxide and hydrogen peroxide (H_2_O_2_), in *Nicotiana benthamiana*, ultimately leading to resistance to the bacterial pathogen *Xanthomonas campestris* (Kim et al., 2013). An Arabidopsis mutant deficient in Spm biosynthesis exhibits hypersensitivity to salt and drought stresses, and the phenotype is mitigated by exogenous Spm, but not Put or Spd (Yamaguchi et al., 2006; Kusano et al., 2007). Together, these findings suggest that Spm is a stress-associated signaling molecule (Yamakawa et al., 1998) due to its unique role in inducing several components of the plant defense response, including: (i) genes coding for pathogenesis related (PR) and resistance (R) proteins (Yamakawa et al., 1998; Gonzalez et al., 2011); (ii) mitogen-activated protein kinases (MAPK) (Takahashi et al., 2003; Gonzalez et al., 2011); (iii) several defense-associated transcription factors (Mitsuya et al., 2009; Gonzalez et al., 2011); (iv) phytoalexin biosynthesis (Marco et al., 2014; Mo et al., 2015); and, (v) the hypersensitive response (HR) (Takahashi et al., 2004; Sagor et al., 2009). In this review, we summarize and integrate current knowledge on Spm-mediated refinement of plant defense responses to both biotic and abiotic stresses, and highlight the importance of Spm as a potent plant defense activator with broad-spectrum effects. In addition, a model is proposed to explain how Spm regulates various oxidative and hormone signaling pathways, which tailor an optimal defense response to various external stresses.

### Spm Metabolism in Plants

Spm anabolism in plants involves two main routes (Shelp et al., 2012). The first is catalyzed by ornithine decarboxylase, which converts ornithine into Put, the main precursor for Spm biosynthesis. The second is a three-step pathway in which arginine is converted to agmatine by arginine decarboxylase, and then agmatine is converted to Put by agmatine imidohydrolase and carbamoylputrescine amidohydrolase. Put is then successively converted to Spd by Spd synthase, and then to Spm by Spm synthase. The latter reactions require the addition of aminopropyl groups, supplied from decarboxylated S-adenosylmethionine (SAM), which is a product of SAM decarboxylase (SAMDC). Spm catabolism involves flavin-containing PA oxidases (PAO), which catalyze two types of reactions, terminal oxidation and back-conversion. The terminal oxidation of Spm generates 4-N-(3-aminopropyl)-4-aminobutanal, 1,3-diaminopropane and H_2_O_2_. Alternatively, the back-conversion reaction converts Spm to Spd, and Spd to Put, resulting in the production of 3-aminopropanal and H_2_O_2_.

## Spm Metabolism and Biotic Stresses

### Spm Induces Oxidative Response

The HR reaction is defined as a type of rapid programmed cell death, which is induced by the generation of reactive oxygen species (ROS, such as H_2_O_2_) at the site of pathogen entry, leading to activation of several defense mechanisms that result in cessation of growth of the pathogen, typically biotrophic, and in protection of remaining plant tissue (Govrin and Levine, 2000; Jones and Dangl, 2006). It is generally believed that the HR reaction is effective against biotrophic pathogens only, but effectiveness of HR against necrotrophic pathogens such as *Botrytis cinerea* has also been reported (Asselbergh et al., 2007; Azami-Sardooei et al., 2010, 2013; Seifi et al., 2013). HR induction involves two major pathways: the host HR is mediated through specific recognition of certain microbes by the surveillance system of the host, namely R proteins (Keen, 1990); and, the non-host HR is non-specific, typically induced in response to a broad spectrum of pathogens in many plants (Heath, 2000). Interestingly, Yoda et al. (2003, 2009) demonstrated that PAO-mediated Spm oxidation strongly contributes to the onset of both host and non-host HRs triggered in tobacco plants by different pathogens, highlighting the importance of Spm catabolism in the regulation of the HR-dependent defense response.

Exogenous Spm induces the expression of several H_2_O_2_-dependent signaling components and transcription factors in Arabidopsis leaves, and results in HR-mediated resistance to cucumber mosaic virus (Mitsuya et al., 2009). The addition of a PAO inhibitor represses the activation of defense genes and alleviates ROS generation and HR, confirming that PAO is involved in the resistance response. Infiltration of tobacco leaf disks with Spm strongly decreases the growth of the biotrophic bacterial pathogen *Pseudomonas viridiflava*, but not the necrotrophic fungal pathogen, *Sclerotinia sclerotiorum*, and co-infiltration of Spm and a PAO inhibitor reverses this protective effect (Marina et al., 2008). Exogenous application of thermospermine, a structural isomer of Spm, induces resistance to *P. viridiflava* in Arabidopsis through PAO-mediated thermospermine oxidation (Marina et al., 2013). Apoplastic Spm accumulates in tobacco plants in response to infection by the (hemi)biotrophic bacterial pathogen *P. syringae* pv. *tabaci*, and PAO overexpression upregulates defense-related marker genes and cell wall-based defense responses, resulting in disease tolerance (Moschou et al., 2009). Similarly, overexpression of a cotton-derived PAO in Arabidopsis results in elevated levels of ROS and resistance to the necrotrophic vascular wilt fungus *Verticillium dahlia* (Mo et al., 2015). The resistance response is mainly mediated by the induction of MAPK and cytochrome P450, culminating in the accumulation of the Arabidopsis-specific phytoalexin camalexin (Mo et al., 2015). Exogenous Spm increases the disease resistance of Arabidopsis against *P. viridiflava*, which is compromised by the PAO inhibitor SL-11061 (Gonzalez et al., 2011). Together, these findings suggest that PAO is a key defense regulator, particularly in response to apoplastically-localized plant pathogens.

### Mitochondrion Membrane Dysfunction

Spm induces apoptosis, a type of programmed cell death, in animal cells through the activation of a group of cell-death-inducing pathways, known as the caspase cascade, which entails the loss of mitochondrial membrane potential and leakage of electron-transfer-chain intermediates, such as cytochrome c, into the cytosol (Moffatt et al., 2000; Stefanelli et al., 2000). Similarly, plant mitochondria are known to play an important role in ROS generation and induction of HR during plant-pathogen interactions (Lam et al., 2001; Hatsugai et al., 2004; Van Breusegem and Dat, 2006). Notably, exogenous Spm induces mitochondrial membrane dysfunction (Takahashi et al., 2003) and the expression of two important defense-associated MAPK, which in turn induce a subset of HR-related genes such as HSR203J (Takahashi et al., 2004). Pre-treatment with bongkrekic acid, an inhibitor of the mitochondrial permeability transition pore, suppresses the induction of HR-related genes, confirming that mitochondrial dysfunction is involved in Spm-induced HR in tobacco leaves (Takahashi et al., 2004).

### Hormonal Regulation

Several HR marker genes, such as HSR203J, are responsive to Spm, suggesting that it is involved in HR induction (Takahashi et al., 2004). These HR markers are also induced in NahG plants, which are highly deficient in the plant hormone salicylic acid (SA), suggesting that Spm-induced HR reaction is independent of the SA signaling pathway (Takahashi et al., 2004). This result is consistent with SA-independent, Spm-induced expression of PR proteins in tobacco (Yamakawa et al., 1998). However, several reports propose a link between JA-associated defense responses and Spm metabolism. For instance, exogenous Spm promotes JA biosynthesis in lima bean (Ozawa et al., 2009), and Spm synthase-overexpressing plants of Arabidopsis have elevated levels of endogenous Spm (two to threefold), resistance to the bacterial pathogen *P. viridiflava*, and expression of components of the JA-dependent defense signaling pathway such as ERF and Myb transcription factors (Gonzalez et al., 2011). Similarly, elevated levels of endogenous Spm in SAMDC-overexpression lines of Arabidopsis are associated with resistance to *Hyaloperonospora arabidopidis* and *P. syringae* and the induction of several defense-associated genes, such as PR and R proteins, as well as genes involved in JA biosynthesis, such as chloroplastic lipoxygenase and allene oxide synthase (Marco et al., 2014). Collectively, these findings suggest that JA signaling positively regulates Spm-mediated defense response to biotic stresses.

## Spm Metabolism and Abiotic Stresses

### Spm Activates Antioxidant Response

Elevated levels of endogenous Spm, as well as the exogenous application of Spm, induce tolerance to various abiotic stresses (Capell et al., 2004; Yamaguchi et al., 2006; Kusano et al., 2007). Fruits of the drought-tolerant tomato cultivar Zarina have elevated levels of endogenous Spm and activities of the antioxidant enzymes superoxide dismutase (SOD) and catalase (CAT), culminating in better tolerance to dehydration-induced oxidative stress (Sánchez-Rodríguez et al., 2016). Similarly, Spm application is associated with higher activities of SOD and CAT in pea plants, mitigating high-temperature-induced chlorophyll degradation (Todorova et al., 2016). Also, Spm induces the tolerance of mung bean seedlings to high temperature, drought or cadmium toxicity, and this is typically associated with elevated activities of SOD, CAT, glutathione S-transferase (GST) and glutathione reductase (GR), and levels of non-enzymatic antioxidants such as ascorbic acid and glutathione (GSH), culminating in reduced ROS accumulation (Nahar et al., 2016a,b). The application of Spm to wheat leaves alleviates oxidative damage caused by cadmium and copper excess, reduces the metal-induced ROS accumulation, and restores GR activity (Groppa et al., 2007). Likewise, Spm application to soybean leaves reduces osmotic-stress-induced losses in chlorophyll, carotenoid and protein levels, and increases the activities of CAT and SOD (Radhakrishnan and Lee, 2013). Stress tolerance, elevated activities of CAT, SOD and peroxidases, and elevated expression of heat shock proteins are found in Spm-treated seedlings of trifoliate orange exposed to combined drought and heat stresses (Fu et al., 2014). Together, this body of evidence suggests that Spm induces tolerance to oxidative stress caused by abiotic stresses through the activation of both non-enzymatic and enzymatic antioxidant pathways.

### Hormonal Regulation

It has previously been shown that exogenous abscisic acid (ABA) upregulates expression of the mPA biosynthesis genes SAMDC and arginine decarboxylase (Urano et al., 2003), and the induction of these genes is significantly compromised in ABA-deficient mutants of Arabidopsis grown under drought stress (Alcázar et al., 2006a), suggesting a positive correlation between mPA biosynthesis and ABA-mediated response to cold, salt and drought stresses (Alcázar et al., 2010). Such a premise is supported by the existence of several abiotic stress-responsive elements (motifs), as well as, ABA-responsive elements in the promoters of mPA biosynthesis genes (Alcázar et al., 2006b). Notably, Spm treatment induces the expression of ABA-responsive element binding factors in trifoliate orange seedlings challenged by drought and heat stresses (Fu et al., 2014). Hence, crosstalk between Spm-mediated defense response to abiotic stresses and ABA-dependent signaling pathway is suggested.

## Contrasting Roles of Spm During Oxidative/Antioxidant Responses

**FIGURE 1:**
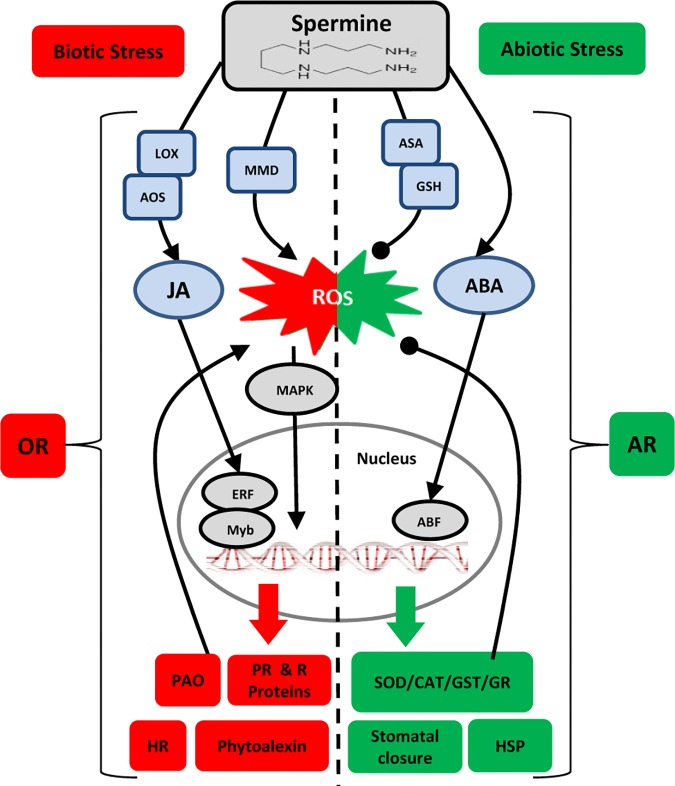
Model for interaction of Spm with plant responses to (a)biotic stresses. Lines ending in arrowheads and closed circles, respectively, indicate positive and negative impacts. Biotic Stress: OR, oxidative response; HR, hypersensitive response; MMD, mitochondrion membrane dysfunction; PAO, polyamine oxidase; JA, jasmonic acid; LOX, lipoxygenase; AOS, allene oxide synthase; MAPK, mitogen activated protein kinase; ERF, ethylene responsive factor; PR, pathogenesis related; R: resistance. Abiotic Stress: = AR, antioxidant response; ASA, ascorbic acid; GSH, glutathione; ABA, abscisic acid; ABF, abscisic acid-binding factor; SOD, superoxide dismutase; GR, glutathione reductase; CAT, catalase; GST, glutathione S-transferase; HSP, heat shock protein.

Many of the key reports on Spm-induced resistance discussed above are summarized in **Table 1**. Examination of the biochemical, transcriptional and molecular responses to (a)biotic stresses leads us to hypothesize dual roles for Spm in modulating the oxidative status of the plant cell. Spm seems to accumulate in response to both biotic and abiotic stresses, but this is followed by two different scenarios: (i) upon perception of biotic challenges, Spm “enhances” the oxidative response through the induction of ROS generation and HR: and (ii) upon perception of abiotic challenges, Spm “alleviates” oxidative damage through the stimulation of ROS-scavenging enzymes, leading to an antioxidant response. **Figure 1** depicts the different players involved in the two scenarios. How a plant adopts such contrasting mechanisms in order to tailor an appropriate defense response merits further consideration.

**Table 1:** Defense mechanisms associated with Spm-induced resistance against biotic and abiotic stresses.

Plant species	Pathogen/environmental treatment	Spm sources	Induction of biochemical, transcriptional or molecular response	Reference
**Biotic Stresses**		
Tobacco (*Nicotiana tabaccum L.*)	Tobacco mosaic virus	Exogenous	MMD; ROS generation; MAPK & HR-related genes such as HSR203J	Takahashi et al., 2003, 2004
Tomato (*Solanum lycopersicum* L.)	Tobacco mosaic virus	Endogenous, exogenous	SA-independent PR proteins such as PR1 & PR5	Yamakawa et al., 1998
*(Arabidopsis thaliana* [L.] Heynh.)	*Pseudomonas viridiflava*	Endogenous, exogenous	R proteins; MAPK; JA-dependent TFs such as Myb & ERF	Gonzalez et al., 2011
Arabidopisis	*Pseudomonas syringae* & *Hyaloperonosposa arabidopsis*	Endogenous, exogenous	PR proteins such as PR1, PR2 & PR5; R proteins (FLS2); JA-biosynthesis proteins such as LOX & AOS; cytochrome P450	Marco et al., 2014
Arabidopsis	*Verticillium dahlia*	Endogenous, exogenous	PAO; ROS generation; MAPK; cytochrome P450; phytoalexin generation (camalexin)	Mo et al., 2015
*Nicotiana benthamiana* L.	*Xanthomonas campestris* pv. *vesicatoria*	Exogenous	ROS/NO generation; HR	Kim et al., 2013
Arabidopsis	Cucumber mosaic virus	Exogenous	PAO; ROS generation; HR; defense-associated TFs such as WRKY40	Mitsuya et al., 2009
**Abiotic Stresses**		
Tomato	Drought	Endogenous	ROS scavenging; enzymatic antioxidant activity such as CAT & SOD	Sánchez-Rodríguez et al., 2016
Mung bean (*Vigna radiata* [L.] Wilczek)	Cadmium toxicity, heat, drought	Exogenous	Antioxidant accumulation such as ASA & GSH; ROS scavenging; antioxidant activities such as CAT, SOD, GST & GR; inhibition of chlorophyll degradation	Nahar et al., 2016a,b
trifoliate orange (*Poncirus trifoliata* [L.] Raf.)	Combined heat & drought	Exogenous	Enzymatic antioxidant activity such as CAT, SOD & peroxidases; heat shock proteins; ABA-responsive-element binding factors	Fu et al., 2014
Pea (*Pisum sativum* L.)	High temperature	Exogenous	Enzymatic antioxidant activity such as CAT & SOD; inhibition of chlorophyll degradation	Todorova et al., 2016
Wheat (*Triticum aestivum* L.)	Cd^2+^ and Cu^2+^	Exogenous	ROS scavenging; activities of antioxidants & antioxidant enzymes such as ASA, GSH & GR; detoxification pathways (degradation of thiobarbituric acid)	Groppa et al., 2007
Soybean (*Glycine max* [L.] Merr.)	Osmotic	Exogenous	Inhibition of lipid peroxidation (i.e., less oxidative stress); enzymatic antioxidant activity such as CAT & SOD	Radhakrishnan and Lee, 2013
Red tangerine (*Citrus reticulata* Blanco)	Dehydration	Exogenous	ROS scavenging; enzymatic antioxidant activity such as SOD & peroxidase	Shi et al., 2010


*Abbreviations: ABA, abscisic acid; AOS, allene oxide synthase; ASA, ascorbic acid; CAT, catalase; ERF, ethylene responsive factors; HR, hypersensitive response; GR, glutathione reductase; GSH, glutathione; GST, glutathione S-transferase; JA, jasmonic acid; LOX, lipoxigenase; MAPK, mitogen-activated protein kinase; MMD, mitochondrion membrane dysfunction; PAO, polyamine oxidase; PR, pathogenesis related; R, resistance; ROS, reactive oxygen species; SA, salicylic acid, SOD, superoxide dismutase; TF, transcription factor.*

The oxidative response occurs immediately after successful recognition of the pathogen by the plant’s surveillance system, following a biphasic pattern (Wojtaszek, 1997). Phase-I consists of a rapid, transient, and low-amplitude burst of ROS generation, occurs within minutes after pathogen recognition, and is known to function as an upstream trigger of several defense-related signaling cascades. Phase-II occurs after few to several hours post recognition, consists of a sustained wave of ROS generation/accumulation of much higher amplitude, and plays a key role in inducing defense-associated genes and HR (Van Camp et al., 1998; De Gara et al., 2003; Torres et al., 2006). While the oxidative response to avirulent pathogens, successfully recognized by the plant’s immune system, generally exhibits a biphasic pattern of ROS accumulation, only phase-I is elicited in response to virulent pathogens that are able to avoid host recognition (Torres et al., 2006). With this in mind, it seems that PAO-mediated ROS generation (i.e., Spm oxidation) during incompatible plant-pathogen interactions exhibits the characteristics of a phase-II oxidative response, as previously proposed (Takahashi et al., 2004). Therefore, it can be posited that Spm oxidation under such conditions is not merely a metabolic feedback mechanism to maintain PA homeostasis, but beyond that, it functions as an important part of the plant immune system to provide the ROS necessary to fuel successful activation of defense genes and formation of HR.

The role of mPA as protective molecular chaperones (Jiménez-Bremont et al., 2014) might explain how Spm induces an antioxidative state in the plant tissue in response to abiotic stresses. The spatial separation of positive charges in PA at physiological pH could enable PA to bind negatively-charged molecules such as nucleic acids, phospholipids and proteins, thereby protecting the structure and function of these macromolecules from degradation and modification (Ruiz-Herrera et al., 1995; Martin-Tanguy, 2001; D’Agostino et al., 2005). This property would also enable the scavenging of free radicals and stabilization of intracellular membranes under stress conditions (Popovic et al., 1979; Groppa and Benavides, 2008; Alcázar et al., 2010; Radhakrishnan and Lee, 2013). This might also explain why mPA are abundant in green, young and actively growing tissues, whereas their titers dramatically decline in senescing organs (Galston and Sawhney, 1990; Del Duca et al., 2000). Considering that Spm contains four nitrogen groups, it could provide greater buffering capacity than Spd and Put (Shi et al., 2010). This is in agreement with previous studies that report exogenous Spm, unlike Spd and Put, has a potent anti-senescence effect on oat and lettuce leaves, as well as Jerusalem artichoketuber (Galston and Sawhney, 1990; Dondini et al., 2003; Serafini-Fracassini et al., 2010). Notably, elevated levels of Spm in an Arabidopsis mutant that lacks the PA back-conversion pathway, are associated with delayed dark-induced senescence, suggesting that Spm is a metabolic defense mechanism against senescence-induced oxidative stress and cell death (Sequera-Mutiozabal et al., 2016).

## Concluding Remarks

Many natural and synthetic compounds are known to activate defense responses against a certain type of stress only, either biotic or abiotic. Those that confer protection against a wide range of both biotic and abiotic stresses are very rare, with silicon being an important exception (Van Bockhaven et al., 2013). In light of the empirical evidence reviewed above, it seems that Spm can be considered as another exceptional molecule with broad spectrum prophylactic effects against both types of stresses. Such effects are exerted through different passive (attributed to the physical and biochemical properties of Spm) and active (attributed to molecular functions of Spm) mechanisms. Given that Spm refines the defense response according to the biotic or abiotic nature of the stress by (i) promoting appropriate hormone-mediated signaling pathways, (ii) modulating oxidative/antioxidant responses, and (iii) inducing several defense-related genes (**Figure 1**), the notion that Spm functions as a plant defense activator becomes more plausible. Nevertheless, several important questions remain regarding these mechanisms. What are the nodes of convergence between Spm-induced signaling pathway and ABA/JA-mediated defense response during (a)biotic challenges? Which specific transcription factors or other transcription-regulating mechanisms control the Spm-induced defense gene activation? What are the regulatory mechanisms that control Spm-mediated oxidative homeostasis during biotic and abiotic stress responses? Considering the immense value of environmentally-friendly methods for plant stress management in sustainable crop production systems, the application of a multidisciplinary approach benefiting from molecular, biotechnological, and breeding strategies seems to be necessary to fully unlock the potential of Spm as a natural plant defense activator with broad-spectrum protective effects.

## Author Contributions

HS conceived and wrote the manuscript. BS supervised the writing and edited the manuscript. Both authors read and approved the final manuscript.

## Conflict of Interest Statement

The authors declare that the research was conducted in the absence of any commercial or financial relationships that could be construed as a potential conflict of interest.
